# “Intersex” Does not Violate the Sex Binary

**DOI:** 10.1177/00243639231155313

**Published:** 2023-05-18

**Authors:** Rashad Rehman

**Affiliations:** 1 Department of Philosophy and Joint Centre for Bioethics, 98586 University of Toronto, Toronto, ON, Canada

**Keywords:** Gender theory, Nonbinary, Sex binary, Transgender

## Abstract

This article supplements Julio Tuleda, Enrique Burguete, and Justo Aznar's “The Vatican opinion on gender theory” (*Linacre*). It supplements their article by providing a stronger argument for the thesis that “intersex” does not violate binary sex in human beings. In their response to Timothy F. Murphy's criticism of “the Vatican's” (rightfully corrected as the Magisterium of the Catholic Church's) position on the sex binary, they argue subsidiarily that “intersex” does not violate the sex binary. However, their argument against Murphy as stated is implausible; however, I provide a much stronger argument for their conclusion that intersex does not violate the sex binary. I intend to perform this supplementation in two stages, assuming the reader's familiarity with “The Vatican opinion on gender theory.” First, I provide a broader background than Murphy's to the challenge that “intersex” conditions violate the sex binary, showing both how Murphy's criticism is unoriginal and how “intersex” both has been, and continues to be, misunderstood. Second, I problematize Tuleda’s argument, and offer the strongest argument for the conclusion that “intersex” does not violate the sex binary on purely secular/nonreligious grounds (addressing Murphy's complaint). I conclude that the Magisterium of the Catholic Church remains correct that sex is binary.

**Summary:**

Julio Tuleda, Enrique Burguete, and Justo Aznar's “The Vatican opinion on gender theory” challenges Timothy Murphy's criticism of sex binarism as endorsed by the Catholic Church. This article strengthens their criticism by focusing on “intersex” conditions.

## Introduction: The Problem, Misunderstandings, and Prevalence Statistics Revisited

Murphy's (2019) thesis that “intersex” challenges the paradigm of sexual dimorphism, the thesis that human beings are exclusively either male or female, is unoriginal.^1^ Here is the challenge within historical context (Delimata 2019):

The struggle to maintain equilibrium is particularly evident where the discursive presumption that sex is dualistic is repeatedly confronted by biological evidence that sex is variant. In the nineteenth and early twentieth centuries the repeated encounters with sex variance led to deeper and deeper explorations of the body in the search for some biological mechanism for maintaining the two-sex system. When this could not be found, psychological theories emerged that deemed it appropriate to surgically ‘correct’ sex variance and lie to patients, thus equilibrium was maintained by rendering sex variance invisible. (p. 80).

Delimata (2019) here points out that as early as the nineteenth century “biological evidence that sex is variant” has continually called into question the “biological mechanism for maintaining the two-sex system.” Murphy's argument sits on the end of a long line of criticism of the sex binary.

Before addressing this criticism, misunderstandings of “intersex” should be avoided. First, intersex individuals are not asexual, but have clear sex biomarkers that makes their sex epistemically uncertain. The biological constitution of intersex individuals involves at least *some* typically male/female biological sex features in their genotype, phenotype, et cetera. Should we place biomarkers aside, it would not be clear what sex *could* mean.

Second, intersex individuals are not hermaphrodites. There are two misunderstandings to be warded off, the first in terms of there being no cases of hermaphroditism in the sense of individuals who are *fully* biological males and females, and the second in terms of embryonic development. Beginning with the former, the reason why “hermaphroditism” is no longer used in medical nomenclature is that there are, simply speaking, no hermaphrodites. While having *both* XX and XY chromosomes is possible, this is not identical to hermaphroditism—for reasons we shall see later in the discussion of the definition of “sex.”^2^ With respect to the latter, Orr's (2020) quotation of Intersex Genital Mutilations: Human Rights Violations of Persons with Variations of Sex Anatomy (2016), the NGO report to the 7^th^ periodic report of France on the Convention Against Torture (CAT). In it, we find the following:

Everybody started out as a hermaphrodite: Until the 7^th^ week of gestation, every fetus has “indeterminate” genitals, two sets of basic reproductive duct structures, and bipotential gonads. Only after the 7^th^ week of gestation, fetuses undergo sexual differentiation mostly resulting in typically male or female sex anatomy and reproductive organs. (p. 32).

However, the *potential*, i.e., hence the use of “ bipotential”, to be male or female is not the same as *actually* being “between” (*inter*sex) male and female. *Bipotential* gonads presexual differentiation are different than *actual* bigonads post-sexual differentiation. Consequently, bipotential gonads do not entail a period of hermaphroditism in prenatal human beings.

Third and finally, Tudela et al. (2020) cite Arcelus et al. (2015) for a prevalence statistic for intersex conditions. The problem is that they cite a prevalence statistic of *transsexual* individuals, not *intersex* individuals—and they are different. Arcelus et al. (2015) defines “transsexual” as “individuals who experience discomfort or distress caused by the discrepancy between their gender identity and the sex they were assigned at birth.” (p. 3). This definition excludes at least two categories of intersex individuals, and consequently cannot be a reliable intersex prevalence statistic. First, it would exclude prenatal intersex individuals, e.g., through genetic screening can reveal whether a child has congenital adrenal hyperplasia (CAH) or androgen insensitivity syndrome (AIS), or during infancy. Second, it would exclude non-gender-dysphoric intersex individuals, and non-gender-dysphoric intersex individuals who did not receive surgery to change sexes. Consequently, even if intersex individuals met the definition of “transsexual” later in life, this prevalence statistic is unreliable. However, I hold that Tuleda et al.'s (2020) point still stands unscathed. Consider Sax's (2010) more reliable prevalence statistic of 0.018 percent. Sax's (2010) estimate carefully corrects Anne Fausto-Sterling's (and others’) statistic of intersex's prevalence being around 1.7 percent. Fausto-Sterling's statistic was based on a mistaken definition of intersex which would include Turner syndrome, Klinefelter syndrome, and late-onset adrenal hyperplasia. In none of these cases is their *true* intersex in which there is sexual uncertainty, i.e., these are not cases of *genuine* sexual ambiguity. As Sax (2010) points out in the 0.018 percent figure, the prevalence is nearly one hundred times lower than Fausto-Sterling suggested.

Fourth (and relatedly), there are misunderstandings regarding the difference between being “sexually atypical” and being “intersex.” Consider Feder's (2014) remark that “the issue of “incidence” of atypical sex has been a vexed one, as it concerns not only the frequency with which children are born with *atypical sex anatomies* but also what counts as atypical sex.” (p. 211ff1, my italics). The idea here is that what it means to be intersex *just is* to have an atypical sex anatomy. There is a plausible case that “intersex” and “atypical sex anatomies” are not synonymous. Having an atypical sex anatomy might refer to the broader category of a difference in one's sexual anatomy, but it does not highlight the heterogeneous property of all intersex conditions: uncertainty with regard to one's sex. As Feder (2014) herself admits, the atypical sex anatomy of “hypospadias” (p. 211ff1) involve children “who are usually regarded as unquestionably male” (p. 211ff1).^3^ However, if atypical sex anatomy does not make a sex-*specific* claim, i.e., saying something about sex *identification*, then it should not be used either as a placeholder for, or as a description of, intersex. Having warded off misunderstandings of “intersex”, I will now provide four arguments that “intersex” does not violate the sex binary.

## “Intersex” Does Not Violate the Sex Binary: Argument Revisited

As I read Tuleda et al. (2020), they argue that “intersex” does not violate the sex binary by way of an *argument from exceptions*.^4^ Their argument goes like this: intersex conditions are *exceptions* to the sex binary, showing only that there are exceptions to a general rule of human beings’ sex—not that human beings’ sex is not either exclusively male or female. Moreover, these exceptions are generally *disorders*, i.e., disorders of sexual development (DSD), disorder implying *a lack of order*, i.e., natural order of human sex = male and female. As disorders and exceptions, the remarkably low prevalence of intersex shows or highlights how there is an obvious natural order in the sex of human beings. There are two problems with this argument. First, it does not provide *positive evidence* for the thesis that sex is binary—it only shows that *most* human beings are male and female (which is not being disputed). While gamete production is later cited, it is leveraged implausibly as an argument, e.g., their argument later is that sex is binary *because* all human life is the result of male and female gametes (again, this would only show that there are males and females, but not that sex is *ultimately* binary). This also gives the mistaken impression that reproductive abilities determine sex—which they do not, as I will argue later. Second, there is equivocation in the use of “disorder.” When the Catholic Church uses “order” (*ordo*), e.g., the “order of love” (*ordo amoris*), it ordinarily refers to God's providential design through natural law in His creation. However, DSD nomenclature uses “disorder” to refer to a *medical* disorder. It is plausible that some intersex conditions are mere differences of sexual development, and some are disorders of sexual development (Rehman, 2022). It is also possible that all intersex conditions are medical disorders and simultaneously present (externally) as “mere differences” (and consequently the “disorder/difference” distinction refers to two distinct phenomena, not two characterizations of the same phenomena). It is not clear, however, whether “intersex” truly refers to “disorder” in the theological sense (that is a question I leave to theologians better suited to discuss such an issue). Given these two problems, there is no successful argument against the claim that “intersex” is a refutation of the sex binary. Here is a stronger, purely secular/nonreligious argument for the claim that “intersex” does not violate the sex binary.

## “Intersex” Does Not Violate the Sex Binary: A Stronger Argument

Here is the full argument in deductive form:

We should begin with the well-known scientific investigations [Assumption 1].
We should define “intersex” in light of “well-known” human sex(es) [Assumption 2].
Since “intersex” is the subject of inquiry, it is not currently “well-known” [From (1) and (2)].
So, male and female sex must be defined in order to understand what “intersex” means [From (1) to (3)].
If sex is defined by gamete production, sex in human beings is exclusively male and female [Disjunctive syllogism].
Sex is defined by gamete production [Antecedent].
(C1) Sex in human beings is exclusively male and female [From (6)].
(C2) “Intersex” does not refute the sex binary [From (1) to (8)].

I will defend (1)–(4) together, as well as defend (5) and (6) together. I will defend (1)–(3) together). The claim of (1) is Aristotle's: “… it is proper to start from the well-known” (*Nicomachean Ethics*, I.IV, s. 5; 1095b). The translation I have used accentuates the plausibility of Aristotle's methodological point. The Greek being translated here is from Bekker's 1831 *Aristoteloge Hoikon Nikomacheion* and reads as follows: *arkteon men oun apo ton gnorimon.* The Greek adjective *gnorimos* is able to be translated as “familiar” or “known”, in addition to “well-known.” I choose the translation of “well-known” here because it accentuates that for Aristotle, it is not enough to be familiar or “know” something to begin with it in one's inquiry: the object in question should be *well-known*, otherwise it is not a proper methodological starting point. Second, failure to appreciate this methodological starting point has monumental consequences. For a book-length treatment of these failures and their consequences, see Henk Ten Have's *Bizarre Bioethics: Ghosts, Monsters and Pilgrims* (2022). As a case-in-point example, see Beauchamp and Childress’ (2013) introduction to their discussion on ‘Moral Status’ (p. 62) where they begin, methodologically by discussing the problem of moral status by disputed, unclear cases. The Aristotelian point is that this methodology is *backwards*: we should begin with the clearest cases of moral status, inquire what their necessary and sufficient conditions are, and then apply it to more difficult cases. (2)–(4) are the application of (1) to our present discussion. In order to apply (4), the crucial and most contentious premise is (5).

I show that (5) is true by arguing that Byrne's (2018a) and Soh's (2020) definition of human sex as defined by gamete production is correct. I include *two* such accounts here because while Byrne's arguments are sound, Soh addresses a potential objection to Byrne's argument and responds to it.

Byrne (2018a)^5^ writes:

Let's start with the biology. Fausto-Sterling's approach to whether some people are neither female nor male is rather indirect. She explains the psychologist John Money's many-fold distinction between chromosomal sex, external genital sex, pubertal hormonal sex, and others. She points out that these do not always align. For instance, there are people who are chromosomally male (XY) but whose external genitalia are female. Fausto-Sterling also notes that Money's “layers of sex” are not themselves binary: there are sex-chromosome combinations other than XX and XY, similarly for the other layers. But where are the original categories of female and male, supposedly the topic of Fausto-Sterling's article? They seem to have disappeared, being replaced by chromosomally-female, genitally-female, and so on…but the question was not whether chromosomal sex is binary, it was whether sex is binary. That question has been evaded, not answered.

The argument here is that “sex” does not refer to the different kinds of classifiable “sexes”, e.g., chromosomal/genetic/phenotypic sex, because each of these refers to *properties* of a given sex but are not *defined* by them. For example, while females are anatomically female, they need not be—as in the case of some intersex conditions. This is unsurprising and explains other facts, e.g., it explains how women are females after having a hysterectomy, and men are still males after being castrated. Moreover, it is precisely by identifying “sex” with these properties that the conceptual mistake is made: sex is not defined by chromosomes, genital appearance, or any other sexual characteristic breakdown; instead Byrne proposes that

The answer has been known since the nineteenth century. As Simone de Beauvoir puts it in *The Second Sex* (the founding text of modern feminism), the sexes “are basically defined by the gametes they produce.” Specifically, females produce large gametes (reproductive cells), and males produce small ones. (Since there are no specifies with a third intermediate gamete size, there are only two sexes.). A glance at the huge variety of females and males across the animal and vegetable kingdoms will confirm that there is nothing else the sexes can be. For instance, the equation female = XX is confused for a fundamental reason having nothing to do with human chromosomal variation: females of numerous species either have different sex chromosomes (as in birds) or else no sex chromosomes at all (as in some reptiles). The XX/XY system is merely the mechanism by which placental mammals like humans typically become female and male; other animals and plants use different means to achieve the same end result. Whenever it is suggested that being female or male is a matter of having certain chromosomes (or primary/secondary sex characteristics), that is a sure sign that the discussion has gone off the rails).

Supplementing Byrne within the present context, this mistake of taking chromosomal sex to be “sex” is found within intersex literature. For example, this is especially found in the following statement in George Wald's “Circumcision” in *Genital Anatomy* (2010): “Every schoolchild knows that femaleness is determined genetically by the possession of two sex or X chromosomes (XX), and maleness by one together with a relatively empty Y chromosome (XY).” (p. 237).^6^ Unsurprising is that this is coupled with a commitment to social constructionism about sex: “What we consider to be male or female is largely cultural in any case; many of our conventional notions in this regard are now in flux and being challenged.” (p. 237).

There is one caveat that needs expression before continuing into understanding Byrne's argument. As Bogardus (2020) points out, it is possible to think that “any human is either male or female and never both” (perhaps for Byrne's reasons) “while still being skeptical that it's always *clear* which of those categories a person is in.” (ff17). In other words, it does not follow that because we cannot tell whether someone is male or female that they are neither or both of them. Of course, this hinges on whether Byrne is right about what it means to be “male” and “female”, and so this requires defense. Back to Byrne's argument, the argument is partially analogical to mammalian sexual differentiation more broadly. His point is that there is nothing special about “human sex” any more than “chimpanzee sex.” As he puts it, “human sex” just means having the property “being human” and having a particular “sex.” While the nonhuman animal kingdom does not share the former property, it does share the latter. He notes the objection that “females and males might not produce gametes” requires the more accurate correction that “females are the ones who have advanced some distance down the developmental pathway that results in the production of large gametes”, e.g., “ovarian differentiation.” And the same point holds for males. He admits there are some cases with true uncertainty, but these are not cases of intersex and they are an even more minuscule population than intersex cases. A consequence of this is that social constructionism about sex is false because, as the nonhuman animal kingdom shows, there is “sex” whether it is nested within a social order or not. However, while his account entails that it is false, he elsewhere argues against it more directly. For the sake of charitability to social constructionism about sex (and to reinforce the plausibility of Byrne's argument made here), here is the argument (2018b):

Now return to the definition of “social construction.” If a category is socially constructed, then in order for an object to belong to the category, the object must exist (or have existed) within a society or social organization. Clearly many animals have belonged to the category *female* (or *male*) without existing within a society of any kind. Indeed, there would have been females and males even if life on Earth had been destroyed by an asteroid half a billion years ago and humans had never evolved. *Female* and *male* are therefore not socially constructed categories; that is, sex is not socially constructed. It might be replied that the slogan “Sex is socially constructed” was supposed to be restricted to humans. *Human* sex is socially constructed, not feline sex or beetle sex. But these replies are confused. The categories of “human sex” are presumably *human female* and *human male*. To belong to the category *human female* is simply to have the property *being human* and the property *being female*; in other words, it is to belong both to the category *human* and the category *female*. But the category *human* is no more socially constructed than the category *female —* and if it were, then the thesis should have been that the category *human* is socially constructed, not that *female* and *male* are socially constructed.^7^

Moreover:

… human children and adults almost invariably exist within social groups, but the occasional human who has always been completely isolated from any society is doubtless part of our history too. (Even if not, there could easily have been such an isolated human.) By the same token, there have surely been socially isolated human females and human males. Perhaps an early hunter-gatherer couple left their group and fended for themselves; she became pregnant, he was eaten by a saber-toothed cat, and she died shortly after giving birth. Their short-lived but developmentally typical child did not exist within any society, but was a human being, and was either female or male.

While Byrne's argument is plausibly analogical, it might also run as a *reductio ad absurdum*. Here are both versions he presents:

1. If sex is socially constructed, then animals not existing within a society are neither male nor female.
2. Sex is socially constructed.
3. Animals not existing within a society are neither male nor female.

The conclusion (3) is clearly absurd: an animal's sex is not defined either by a role or participation in the society in which it lives, nor by human categorization.^8^ The same argument runs for human beings under the bridge-premise assumption that there is nothing special about “human” sex since “animal sex” is really “non-human animal sex”). We get the following argument:

1. If sex is socially constructed, then human beings not existing within a society are neither male nor female.
2. Sex is socially constructed.
3. Human beings not existing within a society are neither male nor female.

Again, (3) is absurd in the same way that it was absurd to deny the affirmation that animals not existing within a society are neither male nor female. There might be more modest ways to understand Byrne's argument. It might be that social constructionism must do, however implausibly, the explanatory work to show how sex for animals is somehow different for human beings, or that there is nothing inconsistent or implausible in affirming (3), or some other strategy. For example, it might be suggested that Byrne is equivocating: There are different senses of “male” and “female” in the human case because we are not just biological organisms akin to animals, and so there is more meaning to “male” and “female” in the human sex case. At best, these replies would show that these terms take on new meanings when conjoined to particular facts about human nature, e.g., that we are social animals and consequently make decisions on how we use biological terms; however, it would not show that sex was a social construct, nor that it was not defined by gamete size. Admittedly this is not explicit from Byrne himself. There might be other ways to interpret his argument. For example, it could be an Inference to the Best Explanation (IBE): If it is true that there are males and females without existing in a social order, then the best explanation of this fact is that sex is not a social construct. This is a plausible rendition of the argument, but this would seem incongruent with Byrne's emphasis on the absurdity of denying the statement “there are males” and “there are females” inasmuch as they do not exist in societies.

Soh (2020) articulates the problem of intersex within the context of the argument that cases intersex constitute a case against there being two sexes, i.e., male and female: “Those advocating that biological sex is a spectrum frequently tokenize the intersex community as evidence for their claims. They will say that because some individuals are born with a mix of sex characteristics, sex is not binary. This is inappropriate for several reasons…[primarily that] sex is determined by gametes.” (p. 24). Here Soh (2020) is in alignment with Byrne that sex is determined by gametes, and this means that social constructionism about human sex is false. This also means that sex is binary. However, like Byrne, Soh uses the case of CAH as a litmus test of the sex-as-determined-by-gametes thesis, but in such a way that addresses a potential objection to Byrne, e.g., that ovotestis is a counterexample to the sex-as-determined-by-gametes thesis:

Intersex people tend to produce one of the two types, or are infertile. The difference lies in the fact that, in some cases, an intersex person's gametes are not in alignment with the sex they identify as. For example, girls with congenital adrenal hyperplasia [CAH] are exposed to unusually high levels of testosterone in the womb. When they are born, they may have genitalia that are ambiguous, such as a clitoris that is longer than average or labia that look like a scrotum. They have ovaries that will produce eggs, typical of girls who are not intersex. But as a result of the masculinization process during development, which influences not only a person's anatomy, but their psychology too, they may identify as male in adulthood. The fact that they have female gametes is not indicative of their sex; however, this is not to say that sex should be categorized as something wholly different if someone is intersex. (p. 24).

Before continuing the CAH example to ovotestis, Soh's argument here appears to create a contradiction since she affirms that “sex is determined by gametes” and that “female gametes are not indicative of their sex”—this seems to imply very explicitly that sex, then, is gametes plus something else, e.g., gametes *plus* self-identification. However, Soh's point here in this present context is not to make a strong claim regarding the legitimacy of the self-identification of sex; rather, it is to point out that *regardless* of one's self-identification, there is a biological reality of sex determined by gametes. Another way of saying this is that regardless of your self-identification, if you have female gametes, then you are a female. She continues:

Regarding whether it would be possible for a person to produce both types of gametes, they would need to possess both ovarian and testicular tissue. Individuals with a condition known as ovotestis do possess such a combination. In most cases, however, only one type of tissue is functional; their ovaries will produce eggs, but their testes are unable to produce sperm. This condition is extremely rare, occurring in 1 in 20,000 births. (p. 25).

The crucial point of Soh here is that “only one type of tissue is functional.” Following Byrne (2018a), this might be taken to mean that they have gone the sexual developmental pathway in one sex far more than the other, resulting in gametes that are not only clearly of the one sex, but which are *functional*. The result would be that sex is determined in most cases by gametes, and in rarer cases by functional gametes. The question then would be making sense of cases in which one has functional male and female gametes: “Regarding the question of whether an individual is capable of producing both, one case study documented a man with ovotestis who was believed by physicians to have produced eggs at one point in time, before his testes began producing sperm… (p. 25). If the case study is right, the statistical rarity is *overwhelmingly* minute. This would be a minute *fraction* of Sax's (2010) statistic of 0.018 percent. However, What Soh does not mention is whether this individual had both *functional* male and female gametes; however, if functional gametes do not do the work of suggesting whether the individual is male or female, then facts about their developmental pathway might do the work. For example, in Iqbal et al. (2011), they provide a case study of an intersex patient with ovotesticular disorder/difference of sexual development (OVO-DSD), “one of the rarest varieties of all intersex anomalies.” (p. 16). Again, functionality aside (no such information was given), they allude to the developmental pathway being dominantly male and consequently raising the child as a boy: “After counselling with the parents it was planned to rear this child as *male due to predominant male phenotype*.” (p. 16, my italics). It is here concluded that (6) is true, and consequently (C1) sex in human beings is exclusively male and female and that (C2) “intersex” does not refute the sex binary. The Magisterium of the Catholic Church remains correct that sex in human beings is binary.

## Conclusion

This article intended to supplement Julio Tuleda, Enrique Burguete, and Justo Aznar's “The Vatican opinion on gender theory” (*Linacre*). It supplemented their article by providing a stronger argument for the thesis that “intersex” does not violate binary sex in human beings. While their argument against Murphy as stated was found to be implausible, I provided a much stronger argument for their conclusion that intersex does not violate the sex binary. I showed this by problematizing Tuleda et al.’s (2020) argument, and replaced it with the strongest argument that holds the conclusion that “intersex” does not violate the sex binary. I did this *on purely secular/nonreligious grounds* (addressing Murphy's complaint) using the arguments of Byrne (2018a, 2018b) and Soh (2020). I conclude that the Magisterium of the Catholic Church remains correct that sex is binary remains true.

## Notes

1. Two notes. First, this view is contrasted with sexual *polymorphism* on which there are many sexes, i.e., more than two sexes. Second, sexual dimorphism is not the thesis that every instance of male and female is known or knowable, but that from the definition of “sex” human beings *can* only be either male or female.

2. Thank you to an anonymous geneticist for pointing out this mistake in my article [reference removed for anonymity] (2022, p. 678). It is false that a person cannot have both 46,XX and 46,XY chromosomes because of chimeras (46,XX/46,XY), the most commonly known genetic cause for ovotesticular DSD. “Chimera” means the early fusion of a 46,XX karyotype zygote with a 46,XY zygote, early in embryonic development. It is similar, although distinct from, mosaicism where a single zygote goes on to harbor distinct cell population with different genotypes.

3. The qualifier of “usually regarded” is very odd since there are no cases of hypospadias among females — it is a male-only condition. However, this might have been intended to include those who self-identify as having a “trans-identity” (but no such in-text indication is alluded to either implicitly or explicitly).

4. I am doing interpretive work here because there is no formal argumentation being made against Murphy (2019) here. Their “argument” must be extrapolated, and I have found three ways to extrapolate their argument.

5. I write Byrne's argument in full in part because I am interpreting Byrne's argument(s). While I think my philosophical reconstructions are charitable, I want to be sensitive to other possible philosophical reconstructions.

6. It is also assumed, in this regard, that it is by holding this view which “avoid[s] biases based on outdated understandings of sex and gender” (InterAct & Lambda Legal, 2018, p. 2) that is supposed to pave the way for intersex health disparities: as Byrne will show, this only gives those against IPS less credibility.

7. One objection to Byrne here is that his view seems to entail that a category can only be socially constructed if the individuals belonging to that category have themselves constructed it—and this is not quite right, e.g., “game animals” is a folk kind which is partly socially constructed. This objection misunderstands Byrne. The point is that in order for there to be a “social construction”, there must be *socially constructing* being done, and without a society or social organization of some kind this is not possible. Consequently, there are no “game animals” independent of human categorization (there are only wild animals, becoming game animals *only* when socially categorized in that way), but there is sex independent of human categorization.

8. While the social constructionist might reply that it is human beings who decide how to use animal differences to divide them into the kinds “male” and “female” (Dasgupta et al. 2020), Lenhart (2015) rightfully points out that sociocultural norms *regarding* “sex” are distinct from “sex” proper. It might be that complicated cases of “sex” call for epistemic humility, e.g., simply remaining uncertain, or choosing sex pragmatically, e.g., choosing a practical sex to navigate the world with, but neither of these *entail* that sex is a social construction—it only follows that what we do with “sex” is in part socially constructed (this point is not disputed). However, there are two points to be made in reply. First, “male” and “female” do explanatory work if sex is defined by gamete production: the difference between “male” and “female” explain why binary creatures have the reproductive faculties that they possess. Second, the sex binarist holds that settling difficult cases of “sex” is not a product of human theorizing; instead, it is a matter of retrieving more information regarding a person's biology to adjudicate whether they are male or female. The social constructionist replies: while the biological features of animals are not impacted by our definitions, *the distinctions we make* can be evaluated along several dimensions. Granting that this is true, consider how we evaluate the distinctions we draw in nature. For example, consider geologists' rock classifications. If I observe a rock and want to distinguish between its separations on its formations and color in ordinary light, I will draw the distinction between its *fracture* and *luster*. The rock objectively (mind-independently) has separations on its formations and color in ordinary light, which is to say that “fracture” and “luster”, referring to those separations, are not socially constructed - the only relevant bit of “social construction” is our language we use to talk about those separations on the formations (what we call “fracture”) and the color in ordinary light (“luster”). This is how biological sex classification works as Byrne describes: some animals objectively (again, mind-independently) have small gametes, and some have large gametes. We then apply terms to distinction, and consequently distinguish “males” and “females” (other languages do similarly i.e., Italian, for instance, uses “maschio” and “femmina”). The only relevant bit of “social construction” is our *language* we used to talk about the animals who produce small gametes (what we call “male”) and large gametes (what we call “female”). We then continually refine our biological categories to ensure they reflect, accurately, what we are referring to - and if there are counterexamples, then we either *refine* those categories (e.g., “male” should include castrated males), or, if we cannot (e.g., if “intersex” were a third sex), then abandoning the categories altogether - or further refining the initial distinction beyond two disjuncts, if necessary. The burden of proof is on those who argue that our categories do not hold up: and I am suggesting in this chapter that *that* argument has not been shown through the example of individuals with intersex conditions.
